# Parenting a child with disability in rural South Africa: Navigating the healthcare system

**DOI:** 10.4102/ajod.v11i0.942

**Published:** 2022-10-25

**Authors:** Marubini C. Sadiki

**Affiliations:** 1 Department of Research Administration and Development, University of Limpopo, Polokwane, South Africa

## Introduction

The United Nations Convention on the Rights of Persons with Disabilities (UNCRPD) recognises the family as the most ‘natural and fundamental’ unit and:

That persons with disabilities and their family members should receive the necessary protection and assistance to enable families to contribute towards the full and equal enjoyment of the rights of persons with disabilities. (Preamble:x [UNCRPD], UN 2006:3)

Disability stakeholders at different levels of society need to recognise the central role of the family in supporting a family member with disability. For children with disabilities, the parents or primary family caregivers usually assume the responsibility of caring for the child and providing all the necessary support (Mckenzie & McConkey 2016). Evidence from research across the globe shows that the parents do not usually receive enough support, especially from healthcare services (which happen to be the first port of call when parents are seeking a diagnosis), therapeutic services and other necessary interventions (Kyeremateng et al. 2019).

In this article, I narrate my first-hand experience of caring for a child with cerebral palsy and navigating the healthcare system as a rural black African single mother from a poor background. I feel compelled to share this story from the vantage point of someone who has progressed from being a rural young mother to being a disability advocate, activist and emerging academic who is more aware of the exclusionary practices experienced by people with disabilities and their families. By sharing my story, I hope that the challenges faced by parents of children with disabilities in accessing health care services, particularly in a rural context, may be understood better by health service providers, policy makers and other relevant disability stakeholders. I acknowledge that my experience of parenting a child with disabilities is not a representation of all mothers of children with disabilities in South Africa or in rural areas, because experiences of parenting a child with disabilities may vary depending on the parent’s contextual and personal factors and the child’s needs (Duma, Tshabalala & Mji 2021). However, having worked with parents of children of disabilities over the last three decades and looking at evidence from literature, I found that there are many commonalities in parental experiences, some of which I shall expound upon later in this article.

## The beginning of my journey: Seeking answers and finding none

In 1988, I gave birth to my first child in the rural Vhembe district, in the Limpopo province of South Africa. My child was born preterm and was in an incubator for a month. When we were discharged from the hospital, I did not know that my child was disabled. After a few months, I noticed that he was not reaching the expected physical developmental milestones like other children of his age. I suspected that something was amiss, and that the health professionals would be the right people to assist. I took him to the nearest hospital and explained the situation to the health professionals. I presented my child’s condition in my local language and the nurse translated for the doctor in English. They told me that all he needed was to do certain exercises and that with time he would be able to walk. I was advised to take him to physiotherapy and occupational therapy every fortnight, but no clear explanation was given of how the therapies were going to help my son. I tried inquiring about the cause of the disability, but I was told not to worry – ‘the child will be fine’ was the answer I received. At first, those words strengthened me, because I had trusted in the healthcare professionals whom I regarded as experts with solutions to my child’s problems. With time, I started feeling frustrated and hopeless because I was told the same thing repeatedly but noticed no progress with my child. He was always in hospital for therapy sessions without any noticeable improvement.

My journey of raising a son with disability became a very lonely space for me as I could not find anyone who seemed to understand what I was going through at a personal and practical level. The staff at the hospital did not seem to have time to answer my questions or to explain things in a way that I could understand. I realised the challenge of being a non-English speaker in a system that did not try to provide information in languages and formats accessible to all. To make matters worse, the father of my child denied the pregnancy; neither was he interested in supporting me and my son after I gave birth. The pain of having to traverse the lonely space was excruciating. I experienced both emotional and physical strain, which I later learnt is a common reality of single mothers caring for children with disabilities (Zuurmond et al. 2018). Due to the absence of support and my lack of understanding of my child’s disability, I suffered from self-blame. I felt ashamed of myself, and I used to ask myself constantly, ‘Why did I give birth to this kind of child? What was the cause? Is it God’s punishment? Am I bewitched?’ These were difficult questions to which I could not find answers.

## Feeling helpless in the hands of healthcare professionals: Power imbalances

Communicating with health care professionals was always a challenge due to the language barrier and the health care professionals’ unwillingness to involve me in my child’s treatment. This made me feel helpless. I was not given an opportunity to share my observations and experience of parenting my child and the knowledge I had gained from taking care of him. I felt I knew much more about his condition and I would have wanted to share my knowledge with the healthcare practitioners, but no room was given for that. I was also afraid to ask questions; I just had to take what I was told to do.

Gona et al. (2018) assert that health professionals underestimate the emotional distress and need for information experienced by parents and carers of children with disabilities. I remember when the physiotherapist gave me pamphlets to read that were written in English with pictures of children with disabilities. I could not read the pamphlet written in English, although I was stressed and desperately seeking for solutions for my child’s disability. The pamphlet was not helpful because I could not understand the language, neither did I have the courage to explain that I was not able to understand the information. I longed for a partnership with the healthcare service providers in which my voice as a mother and caregiver could be valued. Such partnerships are of utmost importance for parent empowerment and to ensure optimal care for the child. When there is a communication barrier between the parent and the healthcare providers, the provision of healthcare proceeds with errors, poor quality and risks to patients’ safety (Schyve 2007).

Kyeremateng et al. (2019) reported that almost all parents of children with disabilities in Ghana visited health facilities to understand their children’s condition, and many were dissatisfied with the explanations given to them by health professionals. I had the same experience. I could not name my child’s condition until he was 3 years old, when I managed to learn this by chance. I was queuing with other parents of children with disabilities at the physiotherapy department when one of the staff members came outside and said, ‘parents of CP children should move from this queue and queue on the other side of the building’. I then knew that my child’s condition was CP. It was a big English word, and I was hearing it for the first time in my life. At that moment, I did not realise the two letters stood for ‘cerebral palsy’.

Every time when people asked me why I was carrying my child on my back going in and out of the hospital, my response was, ‘My child has “CP” problem’. It was the only answer I could give. I did not ask anything because I believed that professionals knew better. Hemming and Akhurst (2009) assert that parents are often faced with a dilemma as a result of the way professionals disclose a child’s condition and the period taken to support the parents in dealing with the challenges that come with having a child with a disability. I experienced this dilemma; for example, I was not sure whether to continue or stop attending therapy sessions because there was no progress.

I developed mistrust towards health professionals which was exacerbated by the emotional pain and sadness of not receiving the support I needed. I felt unimportant in my child’s life and the false hope that I was given disempowered me. It was only later when my child was five years old that I started to seek support from other parents with children with disabilities. Parental support orgnisations equipped me with knowledge and skills to advocate for my son and seek services for him and I am indebted to them until this day.

## Conclusions

This article presented my experiences of parenting a child with disability as a mother living in a rural context. I share this story with full knowledge of the changes that have occurred in South Africa since the end of apartheid in 1994, which include the ushering in of new policies like the *Constitution of South Africa*’s Section 24 which enshrines the right of everyone to an ‘environment that is not harmful to their health or well-being’ (Republic of South Africa 1996), the *White Paper on the Rights of Persons with Disabilities* (Department of Social Development [DSD] 2016) and South Africa’s signing and ratification of the UNCRPD in 2007.

There is, however, recent evidence from different rural South African settings reflecting the challenges of parenting a child with disabilities which have similarities with my own story (Duma et.al. 2021; Mudhovozi, Maphula & Mashamba 2012; Tigere & Makhubele 2019). Tigere and Makhubele (2019) interviewed parents of children with disabilities in Sekhukhune district, a rural area in the Limpopo province, and found that parents are not usually aware of their children’s disabilities, and this has negative effects on their caring responsibilities. Research from other African contexts present similar findings; for example, a study conducted in the Botswana, Malawi and Mpumalanga provinces of South Africa by Booyens, Van Pletzen and Lorenzo (2015) reported that caregivers experienced difficulties in accessing information and services for children with disabilities. In Ghana, Zuurmond et al. (2019) reported that primary caregivers of children with cerebral palsy were unable to access health services, lacked information and were dissatisfied with the support they received from health professionals.

It is imperative for healthcare professionals and parents to work together to support children with disabilities’ acquisition of functional abilities as emphasised by Pedro and Goldschmidt (2019). There is a need for combining parental knowledge and experiences and professional knowledge for the benefit of the child and the family. The lack of communication and partnership with healthcare professionals in my case led to poor progress in my son’s development, as I did not understand the healthcare professionals’ language. Their approach did not motivate me to stimulate my child as I did not see my role in his therapy. Ultimately, both my son and I were disadvantaged by the healthcare system.

I cannot emphasise enough the importance of early diagnosis and early intervention and the provision of information to parents in the process of rehabilitation of their children with disabilities. Appropriate counselling at a grassroots level – for example, at local clinics for those in rural areas – can minimise parents’ emotional challenges. Counselling should be in the local language to ensure adequate and effective communication. Parents also need to be informed about their rights as caregivers of children with disabilities and to be equipped with advocacy skills so that they do not see themselves as passive recipients of services and the professionals as experts who are not to be questioned. Policy makers, healthcare service providers, disability organisations and all the other relevant stakeholders have a role to play in improving the lives of children with disabilities and their family caregivers, especially mothers, who in the South African context usually carry the burden of caring for these children (Duma et al. 2021). More research on interaction of service providers with parents and the parents’ experiences in general is needed in driving the necessary change.
